# Meta-analysis of the prevalence of tuberculosis in diabetic patients and its association with cigarette smoking in African and Asian countries

**DOI:** 10.1186/s13104-018-3390-x

**Published:** 2018-05-15

**Authors:** Fasil Wagnew, Setegn Eshetie, Animut Alebel, Getenet Dessie, Cheru Tesema, Amanuel Alemu Abajobir

**Affiliations:** 1 College of Health Science, Debre Markos University, Debre Markos, Ethiopia; 2 College of Health Science, University of Gondar, Gondar, Ethiopia; 3 Faculty of Medicine, The University of Queensland, Brisbane, Australia

**Keywords:** Tuberculosis, Diabetes mellitus, Cigarette smoking, Systematic review and meta-analysis

## Abstract

**Objective:**

This systematic review and meta-analysis was undertaken to estimate the prevalence of tuberculosis in diabetic patients and to determine the effect of cigarette smoking.

**Results:**

A total of 15 studies was included in the meta-analysis. The pooled overall prevalence of tuberculosis in diabetes was 4.72% (95% CI 3.62–5.83%). In sub-group analyses, the prevalence was 5.13% (95% CI 4.34–5.92%) in Africa, followed by 4.16% (95% CI 2.9–5.4%) in Asia. The odd ratio of tuberculosis among diabetes patients was 7.6 (95% CI 1.46–39) in cigarette smokers as compared to nonsmokers. Publication bias was detected based on graphic asymmetry of fun-nel plots, Begg’s and Egger’s tests (p < 0.05). Tuberculosis is a common co-morbidity in diabetic patients. Tuberculosis-diabetes co-morbidity is significantly higher in cigarette smokers.

**Electronic supplementary material:**

The online version of this article (10.1186/s13104-018-3390-x) contains supplementary material, which is available to authorized users.

## Introduction

*Tuberculosis (TB)* is an infectious disease caused by various strains of mycobacterium, particularly *Mycobacterium tuberculosis,* and usually affects the respiratory system [1]. *Diabetes mellitus (DM)* is a complex metabolic disorder featured by a high level of blood sugar either because of inadequate insulin production or less sensitivity of cells responsible to the insulin metabolism [2], and associated with impairment of cell-mediated immunity involving the lungs, kidney dysfunctions, and micronutrient deficiencies [3]. The World Health Organization (WHO, 2017) reported that there were 10.4 million TB new cases and 1.7 million deaths due to TB [4]. Similarly, 415 million cases and 5.0 million deaths due to DM were registered [5]. Tragically, 95% TB and 75% DM cases exist in low and middle income countries (e.g., Africa and Southeast Asia) [9].

TB and DM co-morbidity is considerably an emerging public health problem [6], and TB is the third leading cause of death among patients with non-communicable disease (NCD), particularly DM [7]. For example, the number of patients with TB-DM co-morbidity is higher than the number of patients with TB-HIV(Human Immuno Deficiency) co-infection globally [8]. That is, the rising prevalence of DM is becoming a challenge to TB control [9, 10] and vice versa. This may partially be due to the risk of uncontrolled hyperglycemia for TB [11] and diabetic [12] patients with substantial immuno compromisation [3]. That said, people with diabetes are three times more likely to develop TB when exposed and approximately 15% of TB globally is thought to be related to diabetes [13]. Moreover, those people with TB and coexisting diabetes have 4 times higher risk of worsening TB treatment outcomes and death during the course of TB regimen [14]. In addition, this may be more complicated by common risk factors for TB including HIV, malnutrition, alcoholism and cigarette smoking [12].

A previous study of TB-DM co-morbidity has reported high prevalence of TB in DM patients, ranging from 1.7 to 36% [9]. As a result, WHO has strongly recommended a collaborative framework for clinical management and control of TB-DM co-morbidity. That is, people with diabetes to be screened for cough of 2 weeks or more at the time of diagnosis for diabetes and, possibly, during regular follow-ups. Thus, three important intervention strategies, namely, establishing mechanisms of collaboration between TB and DM control programs, early detection and management of TB in patients with DM, and early detection and management of DM in TB patients have been recommended [15]. These strategies may also have pivotal roles, notably for high TB burden countries to mitigate the dual burden of TB-DM co-morbidity. Thus, it is crucial to understand the prevalence of TB-DM co-morbidity particularly in low and middle-income countries.

Furthermore, cigarette smoking has adverse effects on respiratory function and is associated with an increased risk of respiratory tract infection and TB-DM co-morbidity [16–19]. Cognizant to this, WHO and International Union Against Tuberculosis and Lung Disease (The Union) have encouraged a National TB Programme to address the combined challenges of smoking, diabetes and TB [20, 21].

Generally, previous TB-DM co-morbidity studies, mainly from African countries, on bi-directional screenings of TB and DM and have not considered the risk of cigarette smoking. In another words, even though some previous studies [22, 23] determined the association between smoking status and the risk of TB in DM patients, the evidence base still remains inconsistence and inconclusive. This systematic review and meta-analysis is therefore aimed to summarize the prevalence of TB in DM adult patients and its association with history of cigarette smoking.

## Main text

### Methods

#### Study design and search strategy

This systematic review and meta-analysis was carried out using both published and unpublished literature to estimate the prevalence of TB among diabetes patients and to determine the risk of TB-DM co-morbidity in cigarette smoking patients. Studies were found through electronic and manual searches using databases, Psych INFO, EMBASE, MEDLINE/PubMed, Google scholar and Google for gray literature from 1980-2017. The search terms were used, entering the following key terms: “prevalence” OR “Epidemiology” AND “tuberculosis,” OR “TB” AND “Diabetes Mellitus,” OR “DM*” OR “Diabet* “Co-morbid*” AND Asian countries” OR “African countries”. Electronic searches were supplemented by screening the reference lists of included studies, expert recommendations and hand searches for sources of gray literature. The preferred reporting of systematic reviews and meta-analysis (PRISMA) guidelines were used [24] for the review.

#### Inclusion criteria

Included studies were those that obtained ethical approval and were undertaken in high TB burden countries (African and Asian countries) and/or reported the prevalence of TB among diabetes patients and/or determine the effect of cigarette smoking in these patients. Peer-reviewed studies with cross-sectional survey or case-series designs, and those studies that involved primary outcome(s) of interest were included.

#### Exclusion criteria

Those studies that reported incidence, only multi-drug resistance TB and latent TB were excluded from the analysis.

#### Data extraction

Two reviewers (FW and SE) screened the titles and abstracts of identified studies and assessed the full text of potentially eligible studies. Any controversy was resolved by consensus. We made some efforts to communicate the authors whenever further information was needed. Data from the included studies were extracted independently by these reviewers. Interestingly, we checked a random sample of 30% of the extracted data and found no difference. Data on author(s), study year, region of study, study design and sample size were extracted using Microsoft excel. The overall prevalence of TB in diabetic patients was also extracted from each included study. Moreover, data on the risk of cigarette smoking among these patients were extracted. AAA critically reviewed the manuscript.

#### Quality appraisal

Articles were assessed for quality score using Newcastle–Ottawa Scale adapted for cross-sectional studies quality assessment tool, with a score of ≥ 5 out of 10 considered as high quality score. Two authors (FW, SE) assessed the quality of each paper. The reviewers compared quality appraisal scores and resolved any disagreements before calculating the final appraisal score. All included studies were of high quality score.

#### Data analysis

Meta-analysis of pooled prevalence of TB in DM patients was carried out using a random-effects model, generating a pooled prevalence with 95% CIs, using STATA/se version 14. Subgroup analyses by continents (Africa and Asia) were carried out because of significant heterogeneity between studies and/or countries.

OR of TB among diabetic patients with cigarette smokers (compared to nonsmokers) was also determined. Heterogeneity among studies was estimated using the I^2^ statistics [25]. Publication bias was determined based on the symmetry of fun-nel plots [26], Begg’s and Egger’s tests [27]. As well, the trim-and-fill analysis was considered to estimate the final effect-size while publication bias detected [28].

## Results

After screening for titles, 1313 studies were excluded because of unrelated topics and duplication. The full-texts of 31 studies were screened and 16 studies of which were subsequently omitted from the meta-analysis for there were insufficient data on outcome(s) of interest. A total of 15 studies representing 23,068 participants, which fulfilled the eligibility criteria, were included in the final meta-analysis (Additional file 1: Figure S1). Sixteen studies were excluded because of 2 studies were reported TB incidence done in Korea and Indonesia [29, 30], 11 studies were not reported outcome of interest [31–41], 1 MDR-TB [42], 2 studies were explore pharmacological aspect of TB-DM [43, 44]).

### Prevalence of TB among DM patients

Among the 15 cross-sectional studies included, 9 (52.6%) were from the Asian countries and the prevalence ranged from 0.38% in Taiwan [45] to 14% in Pakistan [46]. Six (36.8%) prevalence studies were conducted in African countries, and the prevalence revealed as low as 3.4% in South Africa [47] and as high as 6.2% in Ethiopia [22] (Tables 1).

**Table 1:** The descriptive summary of 15 studies on the prevalence of TB among DM patients and its associated with history of smoking in Africa and Asia with high TB burden

Author, year, country	Study region and country	DM	TB	Prevalence (%)	Quality score
Tripathy et al. 1984, India [58]	Asia	219	9	4.1	6
Swai et al. 1990, Tanzania [59]	Africa	1250	70	5.6	7
Feleke et al. 1999, Ethiopia [60]	Africa	1352	78	5.77	6
Qayyum et al. 2004, Pakistan [61]	Asia	95	9	9.47	7
Jabbar et al. 2006, Pakistan [62]	Asia	1458	173	11.8	7
Webb et al. 2009, S.Africa [47]	Africa	258	9	3.48	7
Amin et al. 2011, Pakistan [46]	Asia	100	14	14	8
Kirui et al. 2012, Kenya [63]	Africa	1376	77	5.6	7
Jali et al. 2013, India [64]	Asia	4118	111	2.69	6
Kumpatla et al. 2013, India [65]	Asia	7083	50	0.7	6
Prakash et al. 2013, India [66]	Asia	1670	47	2.8	7
Amare et al. 2013, Ethiopia [22]	Africa	225	14	6.2	8
Lin et al. 2015, Taiwan [45]	Asia	3087	12	0.38	9
Rao et al. 2015, India [67]	Asia	96	10	10.4	6
Tiroro et al. 2015, Ethiopia [23]	Africa	681	26	3.8	8

### Prevalence of TB among DM patients

**Fig. 1:**
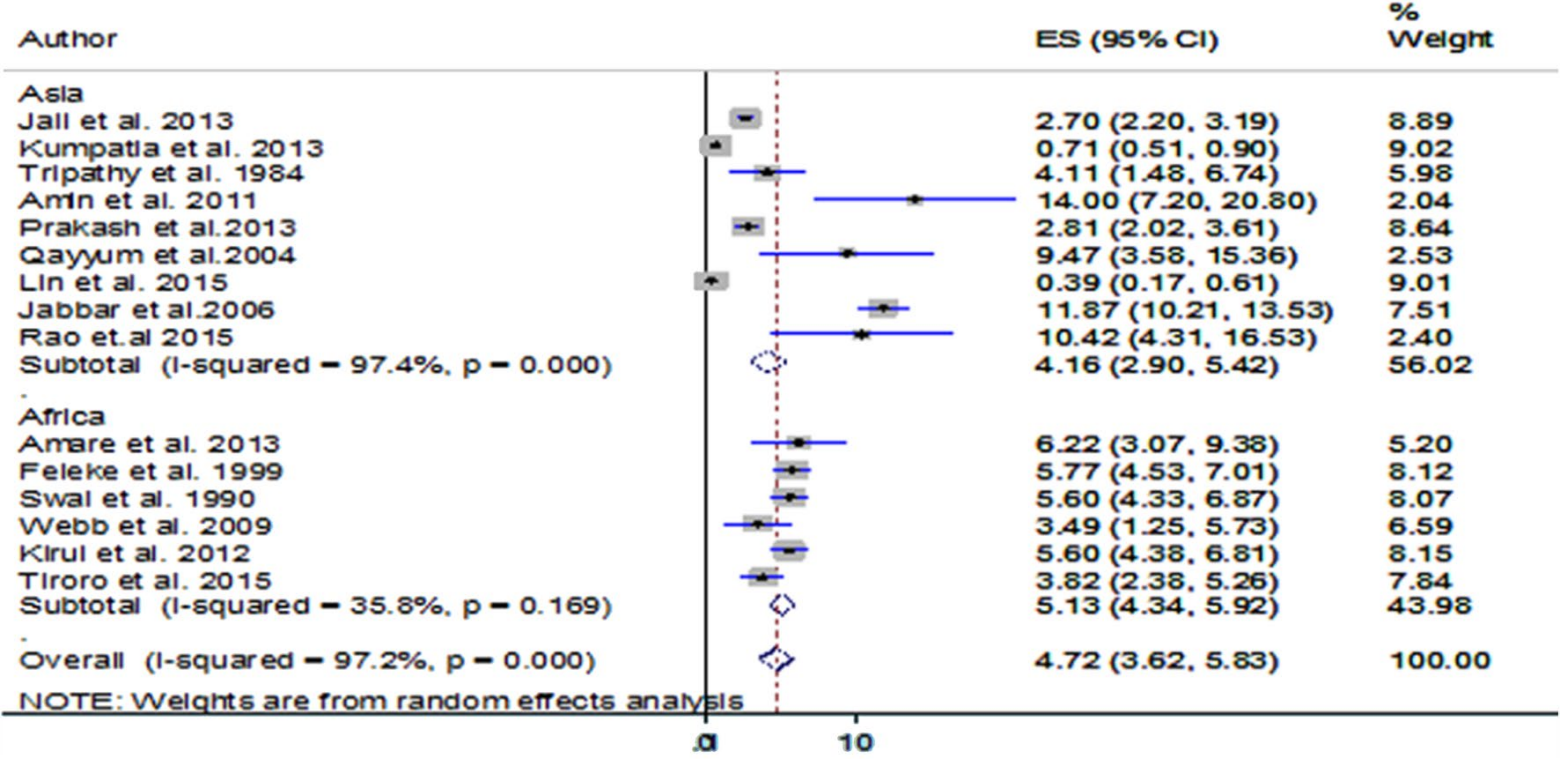
Pooled prevalence of TB among DM patients in African and Asian countries with high TB-burden

The overall pooled prevalence of TB among DM patients was 4.72% (95% CI 3.62–5.83). Based on subgroup analyses by continents, the pooled prevalence of TB among DM patients was 4.16% (95% CI 2.9–5.42) in Asia and 5.13% (95% CI 4.34–5.92) in Africa (Fig. 1).

### Effect of cigarette smoking on TB-DM co-morbidity

**Fig. 2:**
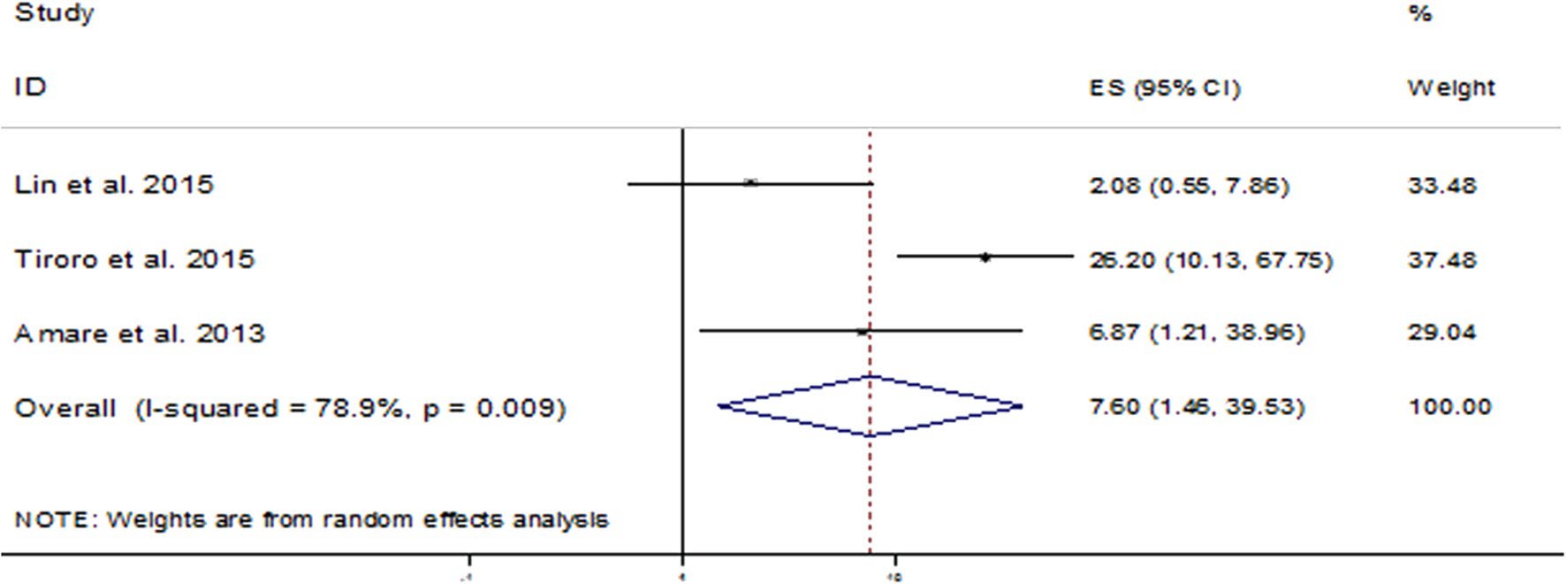
Pooled odds ratio indicating the association of cigarette smoking with TB-DM co-morbidity

To determine the association of cigarette smoking with TB-DM co-morbidity, we included 3 studies which reported data on the prevalence of TB among DM patients who had a history of cigarette smoking. It was shown that the risk of TB-DM co-morbidity was more than 7 (95% CI 1.46–39.53) times higher in patients who had a history of cigarette smoking as compared to their nonsmoker counterparts (Fig. 2).

### Publication bias

Publication bias was detected based on graphic asymmetry of funnel plots, and Egger’s test (p < 0.05). However, Begg’s test indicated no publication bias (Additional file 2: Fig S2). Considering the conflicting evidence on the possible publication bias, trim-and-fill method was used to estimate the publication bias [28]. Though this method indicated 2 potential studies missing, the final results were almost similar to the original findings after 2 virtual studies were appended, indicating that the results of this meta-analysis were steady.

## Discussion

The present meta-analysis was conducted to determine the point prevalence of TB among diabetic patients and its association with cigarette smoking in Asian and African countries with a high TB burden. Existing evidence based on data from 15 included studies with 23,068 study participants revealed a 4.7% point prevalence of TB among diabetic patients in these countries. Poor DM control (as indicated by high HbA1c level) may be associated with differences in the physiological/pathological functions that perhaps boost progression to active TB disease [48] in these patients. This finding is higher as compared to a previous systematic review by Jeon. et al. [9]. The observed variations might be due to aging, changes in lifestyle, and socioeconomic factors. This may be that some countries are experiencing the fastest increase in DM prevalence along with the highest burden of TB and HIV [49, 50]. Despite substantial overlaps of CIs, subgroup analyses by continents suggested the point prevalence of TB among diabetic patients was fairly higher in Africa as compared to Asia. This finding is supported by previous systematic review that reported higher median prevalence of TB in African diabetic patients than the Asian counterparts [51]. This discrepancy might partly be attributable to the functional “Collaborative Framework for the care and control of Diabetes and Tuberculosis” to detect and treat TB in diabetic patients [3] that might have contributed to the lower prevalence of TB-DM co-morbidity. In other words, African countries (e.g., sub-Saharan Africa) have recently been experiencing DM and other chronic conditions [52] possibly because of lack of such an intervention.

A further aim of this study was to determine the effect of cigarette smoking on the occurance of TB among DM patients. Cigarette smoking was found to be significantly associated with TB-DM co-morbidity. This was in accordance with another systematic review that reported exposure to environmental tobacco smoke increases the risks of developing TB disease and infection [53]. Another systematic review by Lonnroth, et al. [54] also revealed that under-nutrition, cigarette smoking and inappropriate alcohol consumption can double or triple the risk of TB-DM co-morbidity. This could be because of the complex etiopathological mechanisms in cigarette smokers resulting in inflammation and oxidative stress that may increase the risk of developing TB-DM [55]. Furthermore, cigarette smoking can increase the availability of iron in lower respiratory tract part [56] that may react with nitric oxide to produce toxic chemicals that can decrease immunity [57].

## Conclusion

TB is a common co-morbidity in diabetic patients. TB-DM co-morbidity is significantly higher in cigarette smokers. Screening for TB in diabetic patients as well as lifestyle intervention may improve early case detection, prevent transmission and decrease the risk of TB-DM co-morbidity.

### Limitations

The inclusion of studies published only in English may compromise representativeness (language bias). Incapability to reliable differentiate between type-I and type-II diabetes mellitus. As well, because of lack of uniformity across each study, we did not explore other factors affecting TB-DM co-morbidity but cigarette smoking.

## Additional files

**Additional file 1: Figure S1.** Flow chart describing selection of studies for a systematic review and meta-analysis of the prevalence of tuberculosis among diabetes patients and its association with cigarette smoking.

**Additional file 2: Figure S2.** Funnel plots, exploring publication bias for the analysis of pooled estimate.

## Abbreviations

DM: diabetes mellitus
TB: tuberculosis
WHO: World Health Organization
