# Supplementary figures and images for: Meta-analysis of the prevalence of tuberculosis in diabetic patients and its association with cigarette smoking in African and Asian countries
Source: BMC Res Notes. 2018 May 15;11:298. doi: 10.1186/s13104-018-3390-x (PMC5952828; doi:10.1186/s13104-018-3390-x)

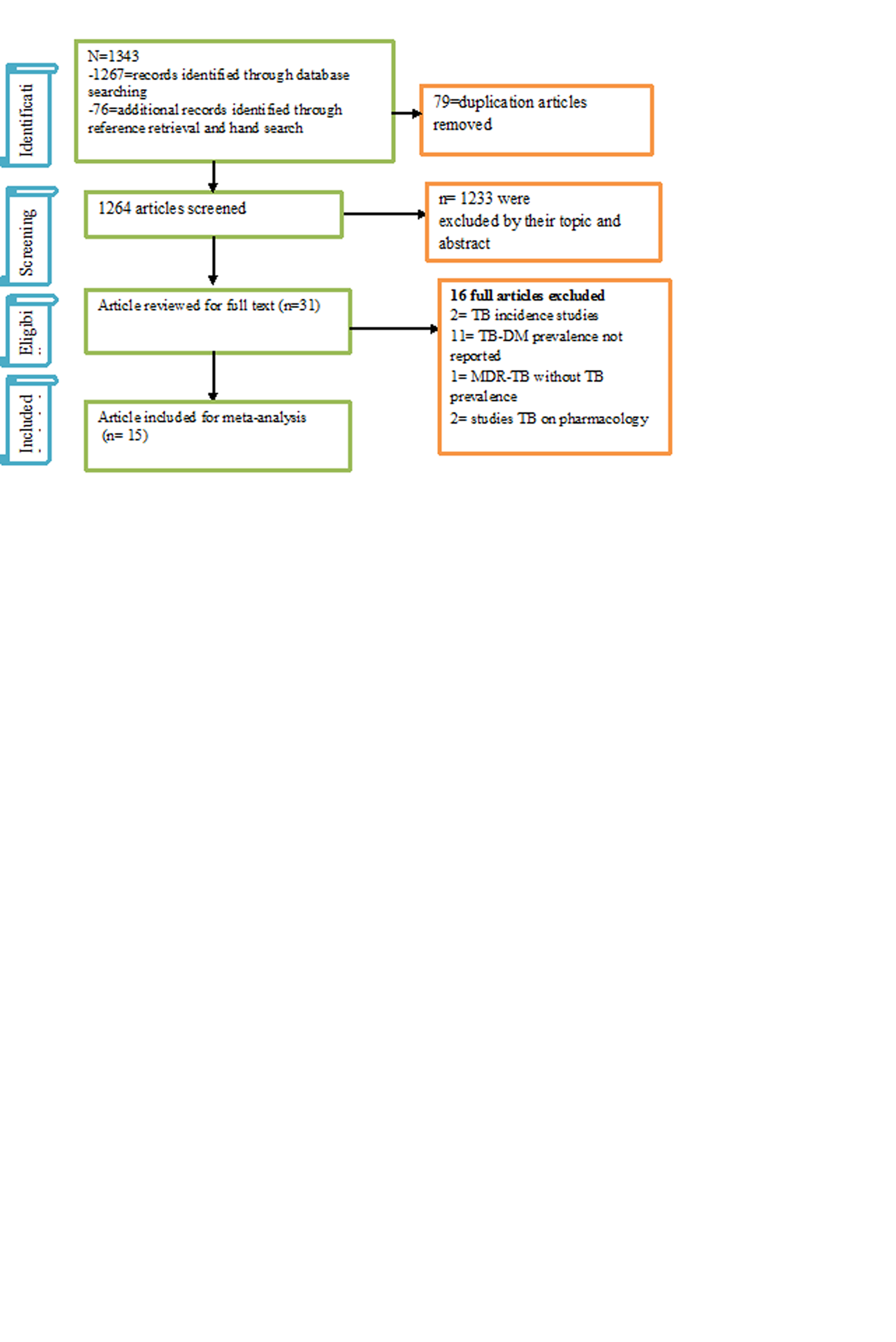

Supplement: Supplementary file 1 — Additional file 1: Figure S1. Flow chart describing selection of studies for a systematic review and meta-analysis of the prevalence of tuberculosis among diabetes patients and its association with cigarette smoking. [file 13104_2018_3390_MOESM1_ESM.png]

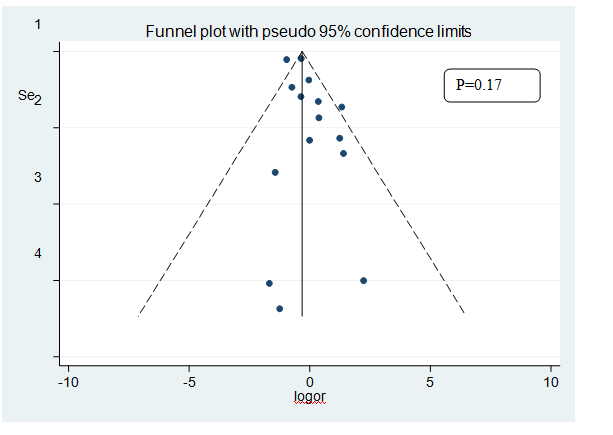

Supplement: Supplementary file 2 — Additional file 2: Figure S2. Funnel plots, exploring publication bias for the analysis of pooled estimate. [file 13104_2018_3390_MOESM2_ESM.png]
